# Synergistic Effect of Carbon Nanotubes and Graphene on Diopside Scaffolds

**DOI:** 10.1155/2016/7090635

**Published:** 2016-04-10

**Authors:** Tingting Liu, Ping Wu, Chengde Gao, Pei Feng, Tao Xiao, Youwen Deng, Cijun Shuai, Shuping Peng

**Affiliations:** ^1^State Key Laboratory of High Performance Complex Manufacturing, Central South University, Changsha 410083, China; ^2^College of Chemistry, Xiangtan University, Xiangtan 411105, China; ^3^Department of Orthopedics, The Second Xiangya Hospital, Central South University, Changsha 410011, China; ^4^School of Basic Medical Science, Central South University, Changsha 410078, China; ^5^Hunan Provincial Tumor Hospital and The Affiliated Tumor Hospital of Xiangya School of Medicine, Central South University, Changsha 410013, China

## Abstract

A synergetic effect between carbon nanotubes (CNTs) and graphene on diopside (Di) scaffolds was demonstrated. 3D network architecture in the matrix was formed through the 1D CNTs inlaid among the 2D graphene platelets (GNPs). The mechanical properties of the CNTs/GNPs/Di scaffolds were significantly improved compared with the CNTs/Di scaffolds and GNPs/Di scaffolds. In addition, the scaffolds exhibited excellent apatite-forming ability, a modest degradation rate, and stable mechanical properties in simulated body fluid (SBF). Moreover, cell culturing tests indicated that the scaffolds supported the cells attachment and proliferation. Taken together, the CNTs/GNPs/Di scaffolds offered great potential for bone tissue engineering.

## 1. Introduction

Diopside (Di, CaMgSi_2_O_6_), as a calcium magnesium silicate ceramic, possesses good bioactivity and cytocompatibility [1]. Ca and Si ions released from Di play an important role in the skeletal and vascular development, which can not only stimulate osteoblasts proliferation and gene expression, but also improve the mineralization of human primary osteoblasts [2, 3]. Mg ion has been recognized to be closely associated with the bone tissue mineralization, indirectly influencing the mineral metabolism [4]. These characteristics of Di make it an attractive candidate biomaterial for bone substitution or regeneration. However, the insufficient strength and toughness hinder its wider applications in some load-bearing conditions. An effective way to overcome this deficiency is incorporating reinforcement phase into the matrix, such as nanometal oxide and carbon nanomaterials.

Carbon nanotubes (CNTs) are 1D carbon nanomaterials with a tube-like structure which possess high strength, good flexibility, and large aspect ratio [5]. Recently, they have attracted much attention of researchers as an ideal reinforcement material in bioceramic composites. It was reported that Zhan et al. fabricated alumina (Al_2_O_3_) nanocomposites with addition of CNTs; the achieved fracture toughness was three times higher than the unreinforced matrix [6]. Chew et al. prepared calcium phosphate cement reinforced with CNTs, and the compressive strength reached the value of 16 ± 4 MPa [7].

Graphene nanoplatelets (GNPs) are composed of a few layers of graphene, with a platelet thickness from 0.34 to 100 nm. The characteristics of GNPs, including their high aspect ratio and large specific surface area, alongside unique graphitized plane structure, determine their potential benefit as ideal reinforcement in the composite materials [8, 9]. Ramirez et al. produced Si_3_N_4_ composites reinforced with GNPs by spark plasma sintering. Remarkable toughening improvement of 135% was obtained compared with Si_3_N_4_ [10]. It is expected that the 2D structures and large surface of GNPs will increase the contact area with the matrix, resulting in improving stress transfer between GNPs and the matrix.

However, CNTs and GNPs tend to aggregate due to the high aspect ratio and strong van der Waals forces, which contribute to the decreased interface between the reinforcement and the matrix [11]. Therefore, the uniform dispersion of the reinforcement is a crucial challenge for effectively enhancing the properties of the composites. Around this issue, it is recognized that the CNTs/GNPs hybrids can promote uniform dispersion of both reinforcements.

In this study, a strategy by combining 1D CNTs and 2D GNPs was explored to improve the mechanical properties of Di scaffolds prepared by selective laser sintering (SLS). Synergetic effect between CNTs and GNPs on the improved mechanical properties of the scaffolds was investigated. Simultaneously, apatite-forming ability, strength degradation behavior, and weight change in simulated body fluid (SBF) were studied in detail to evaluate the bioactivity and degradation. Moreover, the adhesion and proliferation of MG-63 cells were performed to explore the cytocompatibility of the scaffolds.

## 2. Materials and Methods

### 2.1. Materials Preparation

The Di powder from Kunshan Chinese Technology New Materials Co., Ltd., was used as the matrix material. CNTs and GNPs were obtained from Nanjing XF Nano Material Tech Co., Ltd. The Di powder had a particle size of about 200 nm. The CNTs were 1-2 *μ*m in length and 10–20 nm in out diameter. The GNPs were 0.5–2 *μ*m in diameter and about 0.8 nm in thickness. The CNTs/GNPs hybrids were predispersed in acetone with the help of ultrasonic treatment (Kudos, SK3300H, 59 kHz) for 30 min. Subsequently, the hybrids suspensions were incorporated into the Di suspension, which was then sonicated for another 30 min. The produced composites were degassed in vacuum oven at 85°C to eliminate the entrapped air and the solvent. Ultimately, the dry composites were removed from the filter paper and ground thoroughly with pestle. All scaffolds were prepared on a home-developed SLS system using a 100 W CO_2_ laser (model: Firestar® t-Series, Synrad Co., USA), which was introduced in detail in the previous papers [12, 13]. The SLS system consisted of a control system, a laser sintering system, an optical focusing system, and a three-dimensional motion platform. The process parameters of SLS to prepare the scaffolds were set as follows: laser power was 7.8 W, scanning speed was 100 mm/min, and layer thickness was 0.15 mm.

### 2.2. Characterization

The dispersion of reinforcements in the matrix was detected with Scanning Electron Microscopy (JEOL, JSM-6490LA, Japan) following gold sputter coating. Energy dispersive spectroscopy (EDS) analysis was conducted to analyze the elemental constitution of deposits on the fracture surface of scaffolds after immersion in SBF. To confirm the functional groups on the scaffolds after immersion, Fourier transform infrared (FTIR) spectra were recorded with a Thermo Nicolette 6700 spectrophotometer.

Compression tests were carried out by a universal mechanical tester (WD-01, Shanghai Zhuoji Instrument Co., Ltd., China), and the sample dimensions were 3 mm × 1.6 mm × 0.8 mm (*L* × *W* × *T*). Compressive strength was obtained by the maximum stress before failure. Fracture toughness tests were performed by HXD-1000TM/LCD digital micro hardness tester (Shanghai Taiming Optical Instrument Co., Ltd.) with a 300 g force load for 15 s, and an average of five indents was recorded for each sample. To evaluate their mechanical stability, scaffolds were immersed in SBF for 7, 14, and 21 days (see Section 2.3), and then the compressive strength of the scaffolds soaked in SBF was tested.

### 2.3. Weight Loss and Apatite-Forming Ability

The selected scaffolds according to their best mechanical properties were soaked in SBF, to evaluate their bioactivity and degradation behavior. The SBF was prepared on the basis of the procedure proposed by Kokubo and Takadama [14], whose ion concentrations were similar to those in human blood plasma. To prepare the SBF, reagent-grade CaCl_2_, KCl, NaCl, NaHCO_3_, MgCl_2_·6H_2_O, K_2_HPO_4_·3H_2_O, and NaHCO_3_ were dissolved in distilled water and the solution was buffered to pH 7.4 with trimethanol aminomethane-HCl.

To evaluate the degradation of the scaffolds, the initial weight of the scaffolds (*W*_0_) was recorded, followed by immersing in SBF, and the sampling took place after 7, 14, and 21 days. The solution was updated every day. After separating from SBF, the scaffolds were gently rinsed in 10 mL of distilled water for 5 min and reweighed (*W*_1_) after being dried. The weight loss (*W*_*L*_) of scaffolds was calculated as follows: *W*_*L*_ = (*W*_0_ − *W*_1_)/*W*_0_ × 100%, where *W*_0_ and *W*_1_ represent the initial scaffolds weight and the scaffolds weight after immersion, respectively. The apatite-forming ability of the scaffolds was determined with SEM and EDS.

### 2.4. Cell Culture

Human osteogenic sarcoma MG-63 cells were cultured in Dulbecco's modified Eagle medium (DMEM) containing 10% Fetal Bovine Serum (FBS), 1% penicillin/streptomycin, and 1.5% geneticin in standard cell-culture conditions. Before cells seeding, the scaffolds were sterilized under ultraviolet (UV) radiation for 30 min and soaked in ethanol for 15 min at room temperature followed by rinsing with phosphate-buffered saline (PBS). MG-63 cells with a density of 2500 cells/cm^2^ were dispensed onto scaffolds in 12-well plates and cultured for 1, 3, and 5 days. After incubation, scaffolds were removed from the plate and rinsed twice with PBS. The cells adherent to the surface of the scaffolds were fixed with 2.5% glutaraldehyde and dehydrated in 70%, 80%, 90%, and 100% ethanol, respectively, for 2 h.

#### 2.4.1. MTT Assay

Cells viability was studied with an MTT assay by seeding MG-63 cells onto the scaffolds for 1, 3, and 5 days of culture time. At each time point, a 5 mg mL^−1^ MTT solution was added to each well and incubated for 4 h at 37°C. Subsequently, MTT solution was removed and replaced with 500 mL DMSO (dimethyl sulphoxide). After removing the scaffold from each well, the absorbance at 490 nm was measured by a microplate reader (BioTek Instruments Inc., USA).

#### 2.4.2. Fluorescence Techniques

Cell-material interaction was analyzed by fluorescence technique. After the scheduled incubation period, scaffolds were cleaned with PBS, fixed with buffered ice-cold paraformaldehyde (4%), and then permeabilized with 0.1% Tween 20. Then, the cells were rinsed with PBS and preincubated with 1% gelatin in PBS. Subsequently, the cells were incubated in the compound of 4 *μ*M EthD-1 and 2 *μ*M calcein AM in PBS for 30 min. Fluorescence figures were viewed under confocal microscope (Leica Microsystems, Mannheim, Germany).

### 2.5. Statistical Analyses

Statistical analyses were performed using ANOVA with a Scheffé test. A value of *p* < 0.05 was considered to be statistically significant and *p* < 0.01 remarkably significant. All quantitative data were obtained from samples in quintuplicate and were expressed as the mean ± standard deviation (SD).

## 3. Results and Discussion

### 3.1. Dispersion of the Reinforcements

**Figure 1:**
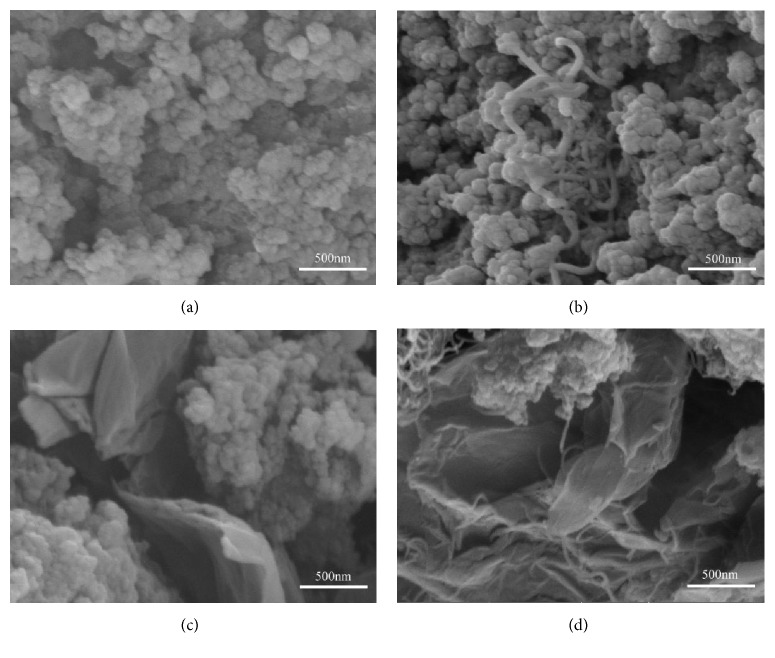
SEM images of (a) pristine Di and composites with (b) CNTs, (c) GNPs, and (d) CNTs/GNPs.

The composite powders were prepared with a total of 2 wt% hybrids (1 wt% CNTs and 1 wt% GNPs). The dispersion states of CNTs and GNPs in the matrix were exhibited in Figure 1. It can be seen that most of the tortuous CNTs were randomly and loosely entangled with each other in the matrix (Figure 1(b)). Meanwhile, composites containing GNPs showed nonuniform dispersion owing to aggregated GNPs (Figure 1(c)). The agglomerates resulted in decreasing the contact area between the reinforcement and the matrix as well as forming structural defects in the matrix, such as holes and voids, possibly limiting the improvement on the composite properties. However, when GNPs were added to the composites with CNTs, uniform dispersion of CNTs was obtained (Figure 1(d)). GNPs improved the dispersion of CNTs owing to their space hindrance effects. At the same time, the CNTs tended to self-align on the surface of GNPs constructing a 3D network structure in the matrix, inhibiting the stacking of GNPs. It led to increasing contact surface area between CNTs/GNPs structures and the matrix.

Other authors have performed similar research. Li et al. prepared epoxy composites with carbon nanotube-graphene nanoplatelet hybrids. With the help of GNPs, highly dispersed CNTs as well as uniform CNTs networks were achieved in the matrix. This led to larger contact area and better interaction between CNTs and the matrix [15]. Chatterjee et al. studied mechanical reinforcement in epoxy matrix with the addition of GNPs and various mixture ratios of CNTs. The CNTs were seen to align themselves on the GNPs creating an interconnected strong nanofiller network in the matrix, thus contributing to the synergistic effect of the hybrids [16]. Consequently, it is anticipated that the synergetic effect between CNTs and GNPs had significant influences on mechanical properties of composites.

### 3.2. Mechanical Properties of Composites

**Figure 2:**
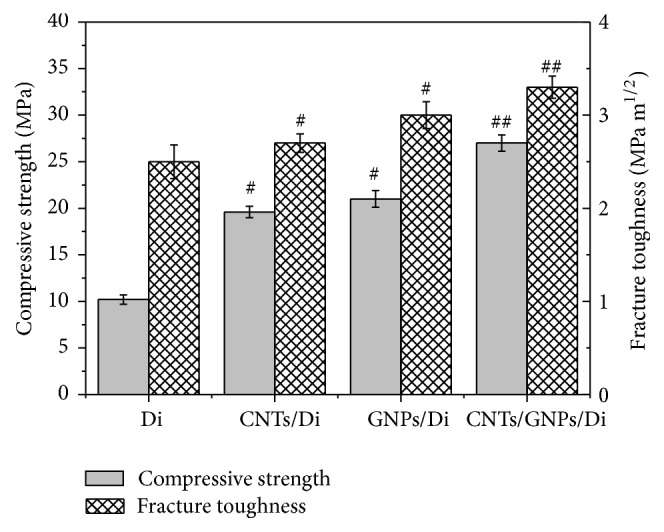
Compressive strength and fracture toughness of Di, CNTs/Di, GNPs/Di, and CNTs/GNPs/Di scaffolds (^#^ *p* < 0.05 and ^##^ *p* < 0.001 compared with Di scaffolds).

To explore the synergetic effect between CNTs and GNPs on the mechanical properties of the scaffolds, compressive strength and fracture toughness of the scaffolds were shown in Figure 2. It was observed that the compressive strength of the CNTs/Di scaffolds (with 2 wt% CNTs) (19.6 ± 0.62 MPa) is about 92% higher than that of Di scaffolds (10.2 ± 0.50 MPa). In contrast, the GNPs/Di scaffolds (with 2 wt% GNPs) exhibited a 105% increase in the compressive strength compared with the Di scaffolds. The variation tendency of fracture toughness with CNTs and GNPs addition was similar to that of compressive strength. The scaffolds reinforced by GNPs presented higher mechanical properties than those reinforced by CNTs. This could be attributed to the contact geometry of 2D surface contact in GNPs/Di scaffolds which exhibited a higher contact area between matrix and the reinforcement, compared to the 1D linear contact in CNTs/Di scaffolds, which resulted in the improvement of interfacial stress transfer between reinforcement and matrix in GNPs/Di scaffolds compared with CNTs/Di scaffolds.

When two reinforcements were brought together into the matrix, CNTs/GNPs/Di scaffolds acquired significant increases in compressive strength and fracture toughness compared with scaffolds with individual CNTs or GNPs, reaching their maximum values of 27 ± 0.88 MPa and 3.3 ± 0.12 MPa·m^1/2^, respectively. This might be owing to the synergetic effect of CNTs and GNPs. CNTs bridged adjacent GNPs to form 3D network structure, which inhibited agglomeration of GNPs, thus increasing the contact surface area between CNTs/GNPs and the matrix. Moreover, CNTs acted as chelating arms for the 3D network structure, which could provide stronger interaction between CNTs/GNPs and the matrix. Therefore, the combining of 1D CNTs and 2D GNPs would become a very significant concept for superior mechanical performance compared with the scaffolds reinforced by CNTs or GNPs alone.

The scaffold fabricated in our study possessed interconnected pore structure which was ~600 *μ*m in *X*-*Y* plane. The dimension of the scaffold is 15 × 15 × 5 mm^3^. The scaffolds were composed of pores and struts and the strut was dense. The porosity of the porous scaffold was measured by water displacement using the Archimedes method. In order to measure the porosity, the apparent volume and true volume of the scaffolds were calculated. The apparent volume was calculated as 1125 mm^3^ (apparent volume = length × width × height) based on the geometry. The true volume was measured in water, which was 474 mm^3^. Porosity of the scaffold could be estimated as 57.9% by

(1) $$\begin{array}{c} P=\frac{\left(V_{a}-V_{t}\right)}{V_{a}}\times 100\%, \end{array}$$

where the porosity, apparent volume, and true volume were designated as *P*, *V*_*a*_, and *V*_*t*_, respectively.

### 3.3. *In Vitro* Bioactivity Tests

**Figure 3:**
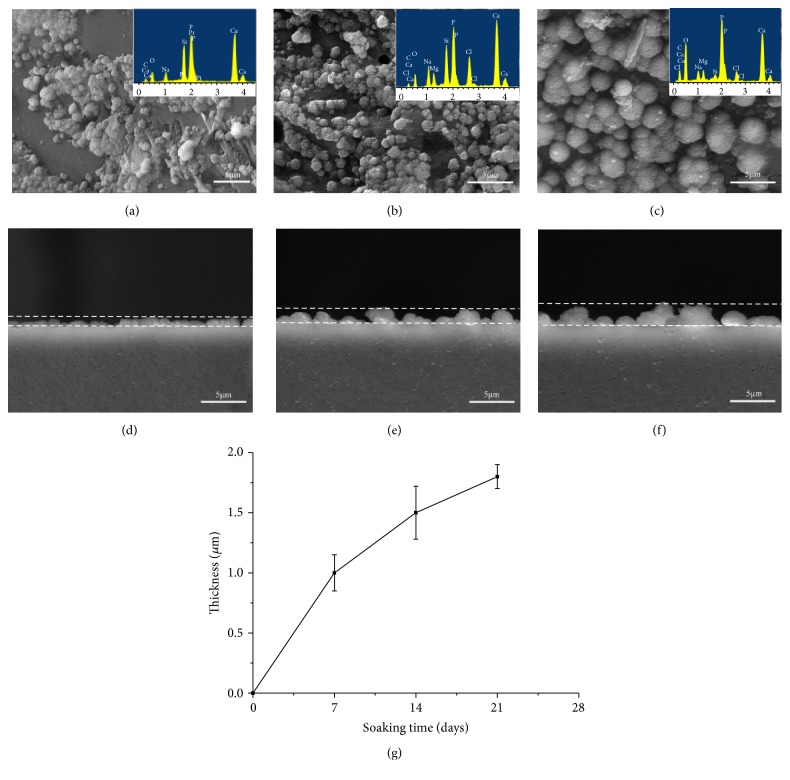
Surface morphologies of the scaffolds after immersing for (a) 7 days, (b) 14 days, and (c) 21 days; cross-sectional view of the scaffolds for (d) 7 days, (e) 14 days, and (f) 21 days and (g) changes in apatite layer thickness on the scaffolds as a function of soaking time.

CNTs/GNPs/Di scaffolds with optimal mechanical properties were chosen to investigate the bioactivity and cytocompatibility. The surface morphologies of the scaffolds after various immersion intervals (7, 14, and 21 days) were shown in Figure 3. It was demonstrated that, after 7 days of immersion, spherical particles were evenly formed on the scaffolds (Figure 3(a)). After 14 days, spherical particles grew gradually and formed a compact layer (Figure 3(b)). After 21 days of immersion, the scaffolds were completely covered by particles with typical worm-like morphology (Figure 3(c)). Composition of the scaffolds after SBF immersion was tested by EDS analysis. It was noted that as the immersion time was prolonged, Si and Mg contents decreased and P contents increased, leading to the decrease of Ca/P ratio. The ratio reached 1.70 after 21 days, closing to that of HAP (1.67), suggesting that the precipitation of apatite occurred on the scaffolds.

In addition, in order to evaluate the thickness increase of formed apatite layers, cross sections of the scaffolds after soaking in SBF for 7, 14, and 21 days were observed (Figures 3(d)–3(f)). The thickness of apatite layers was indicated by an imaginary line. It was noted that the layer thickness increased steeply and reached approximately 1 *μ*m in 7 days. Subsequently, the overall reaction rate decelerated with increasing immersing time. The apatite layer thickness reached 1.5 *μ*m at 14 days and 1.8 *μ*m at 21 days. Accordingly, the reaction rate during the 7 days of immersing was about 0.14 *μ*m per day compared with 0.07 *μ*m per day during the 14 days and 0.04 *μ*m per day during the 21 days of immersion. It was seen that the change of the reaction rate corresponded well with the formation morphology of apatite layer and thickening of the layer. The slowing down of the formation rate could be attributed to the consumption of P ions from the SBF solution. It was prospected that as long as the ion exchange between the scaffolds and SBF happens, the new apatite layer would continually form. The process would end when the provision of P ions from the SBF stopped or when the ions diffusion across the interface is terminated due to the thickness of new apatite layer arriving at a critical value.

**Figure 4:**
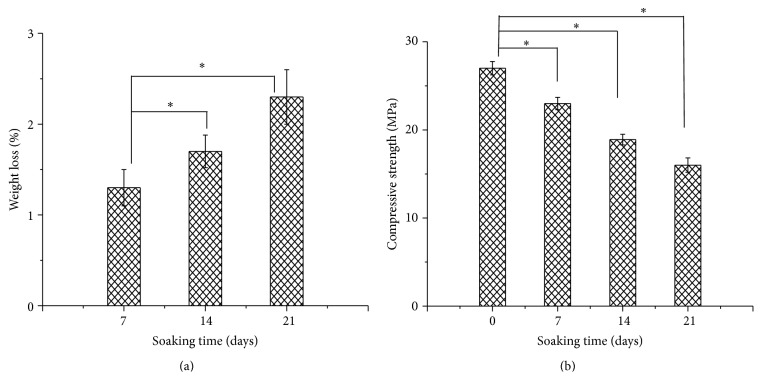
The change of (a) weight loss and (b) compressive strength of the scaffolds as a function of immersion time in SBF (^*∗*^ *p* < 0.05).

To evaluate degradation behavior and mechanical stability of the scaffolds, weight loss and compressive strength after soaking in SBF were shown in Figure 4. It was shown that the weight loss increased with the immersion time increasing and reached 2.3% after 21 days. Meanwhile, the compressive strength of the scaffolds decreased with the immersion time increasing due to degradation. The compressive strength of the scaffolds decreased only by 30% after immersing for 14 days. The decreasing rate was lower than those of HAP and CaSiO_3_ scaffolds [17, 18], indicating that the scaffolds had improved mechanical strength as well as enhanced mechanical stability compared with these ceramics. This could be attributed to the existence of Mg atom in Di. In general, Mg could occupy the position of Ca, forming Mg-O bond which was stronger than the Ca-O bond in HAP and CaSiO_3_; therefore, a more stable crystal structure was formed.

**Figure 5:**
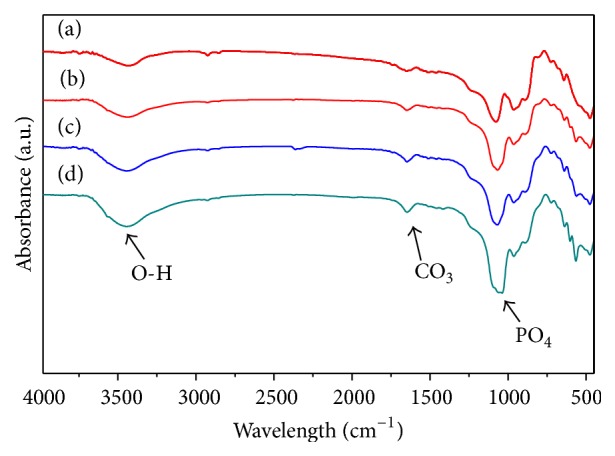
FTIR spectra of the scaffolds (a) before and after soaking in SBF: (b) 7 days, (c) 14 days, and (d) 21 days.

The FTIR spectra of the scaffolds before and after immersing for 7, 14, and 21 days were shown in Figure 5. The spectrum of the scaffolds before immersing (Figure 5(a)) was dominated by silicate absorption band at around 1010, 972, 930, 850, 639, and 586 cm^−1^ [19]. Up to immersion time of 7 days, the scaffolds revealed new stretching peaks for the P-O at 1050, 610, and 570 cm^−1^, ascribed to the formation of calcium phosphate. Besides, C-O and O-H absorption bands appeared at the peaks of 1400 cm^−1^ and 1600 cm^−1^ [20], respectively, which indicated that apatite was formed on the scaffolds in SBF. With immersion time increased, the intensity of the silicate absorption peaks weakened and carbonate absorption peaks increased. All indicated that the scaffolds exhibited excellent apatite formation ability.

### 3.4. Cytocompatibility

**Figure 6:**
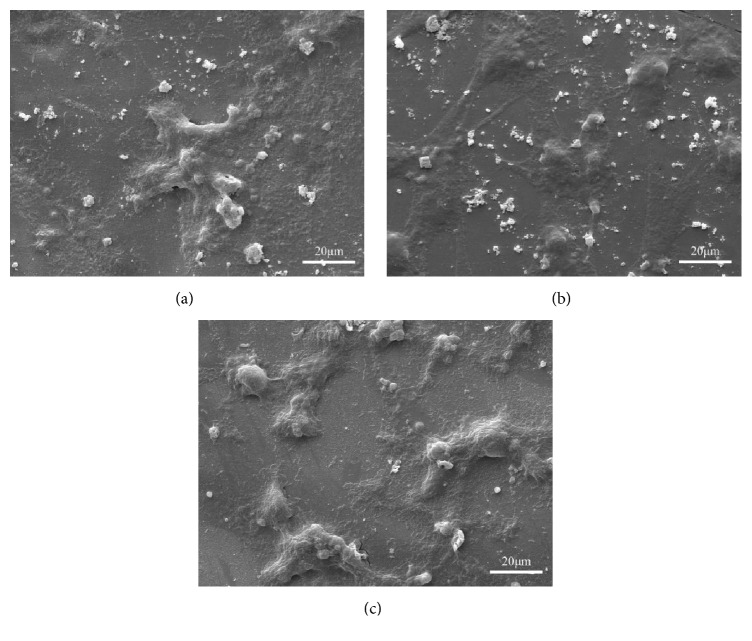
The morphology of MG-63 cells seeded on the scaffolds after (a) 1, (b) 3, and (c) 5 days.

CNTs/GNPs/Di scaffolds with optimal mechanical properties were chosen to investigate the cytocompatibility. The morphologies of the cells on the scaffolds were shown in Figure 6. After culturing for 1 day, flat cells actively spread with filopodia on the scaffolds, which was an obvious indication of the cell attachment and growth process (Figure 6(a)). After culturing for 3 days, large numbers of the proliferating cells with thin cytoplasmic digitations were well dispersed and in close contact with the scaffolds (Figure 6(b)). With the culturing time increased to 5 days, cells continued to grow, aggregate, and cluster till forming confluent layers on the scaffolds (Figure 6(c)), suggesting the good cytocompatibility of the scaffolds.

**Figure 7:**
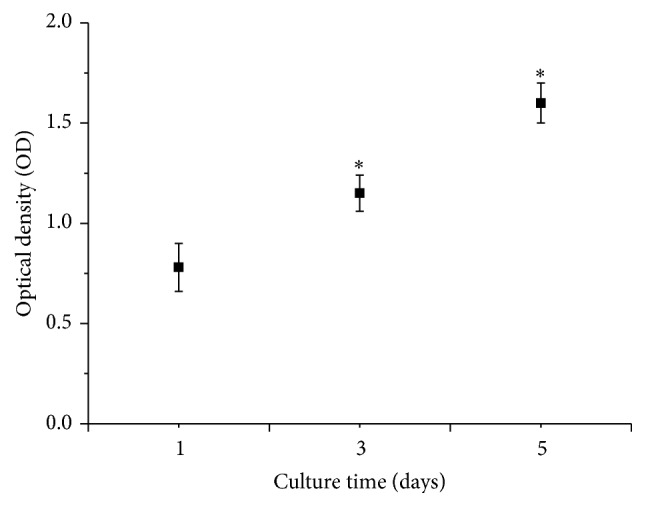
MTT assay of MG-63 cells seeded on the scaffolds for 1, 3, and 5 days (^*∗*^ *p* < 0.05 compared with scaffolds culturing for 1 day).

**Figure 8:**
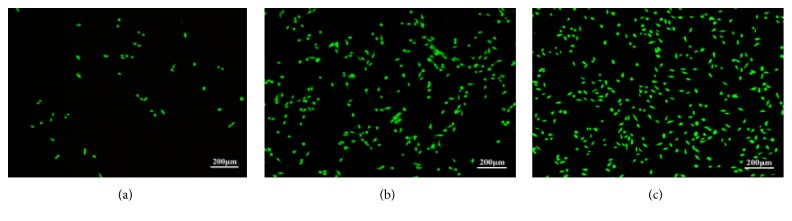
Viability assays of MG-63 cells after different days cultured on the scaffolds: (a) 1 day, (b) 3 days, and (c) 5 days.

MTT assay was demonstrated in a cell viability curve with different time of culture. MTT activity increased with culture time, indicating that the number of cells increased with the culture time (Figure 7). In addition, fluorescence technique was used to determine the cytotoxicity of the scaffolds. Fluorescence figures of MG-63 cells seeded on the scaffolds after different days were shown in Figure 8. It was revealed that the viable cells were green, were well spread, and were distributed throughout the scaffolds. More cells were proliferated on the scaffolds with the increase of culture time. All indicated that CNT/GNPs/Di scaffolds were suitable for MG-63 cell proliferation.

## 4. Conclusions

In this study, compressive strength and fracture toughness of CNT/GNPs/Di scaffolds prepared using SLS were improved by 164% and 32%, respectively, compared with those of Di scaffolds. Notably, the reinforcing effect of the CNT/GNPs hybrids outperformed those of individual CNTs and GNPs. This was attributed to the distinctive 3D network architecture of the CNT/GNPs hybrids. Moreover, the weight loss of the scaffolds in SBF increased lineally, and the compressive strength of the scaffolds decreased by 34% after immersing for 14 days, indicating that the scaffolds possessed moderate degradation rate and reliable mechanical stability. Furthermore, the scaffolds demonstrated good bioactivity and cytocompatibility in terms of apatite formation as well as MG-63 cells attachment and spreading.
